# Voice Stress Analysis: A New Framework for Voice and Effort in Human Performance

**DOI:** 10.3389/fpsyg.2018.01994

**Published:** 2018-11-20

**Authors:** Martine Van Puyvelde, Xavier Neyt, Francis McGlone, Nathalie Pattyn

**Affiliations:** ^1^VIPER Research Unit, LIFE Department, Royal Military Academy, Brussels, Belgium; ^2^Brain, Body and Cognition, Experimental and Applied Psychology, Department of Psychological and Educational Sciences, Vrije Universiteit Brussel, Brussels, Belgium; ^3^Clinical and Lifespan Psychology, Department of Psychological and Educational Sciences, Vrije Universiteit Brussel, Brussels, Belgium; ^4^School of Natural Sciences and Psychology, Faculty of Science, Liverpool John Moores University, Liverpool, United Kingdom; ^5^MFYS-BLITS, Department of Human Physiology, Vrije Universiteit Brussel, Brussels, Belgium

**Keywords:** voice stress analysis, stress, human performance, voice output, bottom-up and top-down modeling, Model for Voice and Effort

## Abstract

People rely on speech for communication, both in a personal and professional context, and often under different conditions of physical, cognitive and/or emotional load. Since vocalization is entirely integrated within both our central (CNS) and autonomic nervous system (ANS), a mounting number of studies have examined the relationship between voice output and the impact of stress. In the current paper, we will outline the different stages of voice output, i.e., breathing, phonation and resonance in relation to a neurovisceral integrated perspective on stress and human performance. In reviewing the function of these three stages of voice output, we will give an overview of the voice parameters encountered in studies on voice stress analysis (VSA) and review the impact of the different types of physiological, cognitive and/or emotional load. In the section “Discussion,” with regard to physical load, a competition for ventilation processes required to speak and those to meet metabolic demand of exercised muscles is described. With regard to cognitive and emotional load, we will present the “Model for Voice and Effort” (MoVE) that comprises the integration of ongoing top-down and bottom-up activity under different types of load and combined patterns of voice output. In the MoVE, it is proposed that the fundamental frequency (F0) values as well as jitter give insight in bottom-up/arousal activity and the effort a subject is capable to generate but that its range and variance are related to ongoing top-down processes and the amount of control a subject can maintain. Within the MoVE, a key-role is given to the anterior cingulate cortex (ACC) which is known to be involved in both the equilibration between bottom-up arousal and top-down regulation and vocal activity. Moreover, the connectivity between the ACC and the nervus vagus (NV) is underlined as an indication of the importance of respiration. Since respiration is the driving force of both stress and voice production, it is hypothesized to be the missing-link in our understanding of the underlying mechanisms of the dynamic between speech and stress.

## Introduction

Speech is one of the most complex but also one of the most important of human motor skills. We use speech to inform others about our environment and to exchange ideas about our current physical and mental state, both in a personal and professional context. In our common daily communication, speech is non-verbally supported. However, in some conditions, people need to rely on speech only, and this in sometimes crucial and stressful circumstances (e.g., professional radio communication). Both voice production and processing rely on the cooperation of approximately 100 muscles, innervated by a diverse network of cranial and spinal nerves (Duffy, 2000) as well as subcortical and cortical parts of the brain (Jürgens, 2002; Carlson, 2016) and cardiorespiratory processes (Câmara and Griessenauer, 2015). Therefore, it is not surprising that speech often deteriorates during situations of challenged human performance or emotional dysregulation. Hence, speech is a psychophysiological process, influenced by environmental and/or internal challenges (Hansen and Patil, 2007). Because of this complexity, the analysis of subtle events in the voice may offer a window into the impact of stress in situations where human performance needs high functioning, but where environmental and/or task-related circumstances make it difficult to monitor stress effectively.

The idea of voice stress analysis (VSA) (For a glossary of abbreviations in the current paper, see Table 1) has already led to the development of some application devices. For instance, in forensic psychology, the technique has regularly been tested to detect lies and deceptions by means of microtremor detection (e.g., Vrij, 2008; Kirchhübel et al., 2011). However, the reliability of the devices used in this type of VSA is a matter of debate (e.g., Horvath, 1982; Giddens et al., 2013). Not only the reliability of the ‘lie-detector,’ but also the fact that the success is dependent on the examiner’s experience, are making the method largely questionable (e.g., Hopkins et al., 2005). Moreover, it has been shown that guiltless persons, whose innocence is challenged, display at least as much stress as guilty individuals, increasing the risk on false alarms (Vrij, 2008). In a recent review (Giddens et al., 2013), it was concluded that, although VSA could be considered as a promising stress detection tool, large interindividual differences are problematic. However, as pointed out in other papers, interindividual differences may just be the key to understanding the functional reactivity of an organism when not seen as errors but, on the contrary, as a signal (e.g., Cain, 2007; Grassmann et al., 2017). Voice output is a psychophysiological response that is part of the human integrative psychophysiological stress system (e.g., Thayer and Lane, 2000, 2009) and stress reactivity is the complex integration of sympathetic and parasympathetic control, occurring in co-activation/co-inhibition (i.e., a simultaneous increase/decrease), reciprocity (i.e., one increases whereas the other decreases) or independent from one another (i.e., either sympathetic or parasympathetic) (Berntson et al., 1991). Moreover, the respective activation and/or deactivation of each of these responses is dependent of individual features such as the environmental challenges one is confronted with along with his or her anxiety-traits and stress-coping mechanisms (Hancock and Szalma, 2008; Thayer and Lane, 2009). Consequently, one of the challenges of psychophysiological research is to understand the dynamic interactions between the different physiological components to gain a clear understanding of these interindividual differences and their underlying mechanisms of autonomy control (Berntson et al., 1991; Grossman et al., 1991). Similarly, the challenge of VSA is probably not to find a universal voice parameter for stress, but, rather, to understand the link between certain parameters and their underlying mechanisms of autonomic control.

**Table 1:** Glossary of abbreviations.

Abbreviation	Name	Description
ACC	Anterior cingulate cortex	The frontal part of the cingulate cortex.
ANS	Autonomic nervous system	One division of the peripheral nervous system, being part of the nervous system.
AR	Articulation rate	SPP divided by the total length of the sample minus the duration of pauses.
BP	Blood pressure	Pressure of the blood measured in the arteries.
CNS	Central nervous system	Part of the nervous system that contains the brain and spinal cord.
EEG	Electroencephalography	The electrophysiological monitoring of electrical activity of the brain.
GSR	Galvanic skin response	The monitoring of electrodermal activity as a reflection of sympathetic activity.
HNR	Harmonic to noise ratio	Indicator of the amount of periodicity against aperiodicity in the voice
HR	Heart rate	The number of heartbeats per unit of time.
HRF	Harmonic richness factor	The ratio of the sum of the amplitudes of the harmonics and the amplitude of the component at the fundamental frequency.
HRV	Heart rate variability	The natural variability in the heart rate under influences of the autonomous nervous system.
IP	Inappropriate pauses	Number of inappropriate breathing pauses within one phrase.
MFCC	Mel-frequency cepstral coefficients	The coefficients that make up the Mel-Frequency Cepstrum.
NAQ	Normalized amplitude quotient	Indicator for breathiness of the voice: ratio of the maximum peak-to-peak amplitude of the glottal flow to the minimum of the glottal flow derivative, normalized by the fundamental period and the sampling frequency.
NV	Nervus vagus	Cranial nerve X.
OCQ	Open and closing quotients	Timing of opened and closed phases of glottal waveform.
SNR	Signal to noise ratio	Indicator of the amount of periodicity against aperiodicity in the voice.
SPP	Syllables per phrase	Number of used syllables between two inspirations.
STAI	State-Trait Anxiety Inventory	Questionnaire to measure anxiety states and traits.
VO_2_/VO_2_max	Oxygen consumption/maximal oxygen consumption	Oxygen consumption/the maximum volume of oxygen the body can consume during intense exercise.
VOT	Voice onset time	The time interval between the release of a plosive such as ‘p,’ ‘b’ and the beginning of the vocal fold vibration associated with the subsequent vowel.
VSA	Voice stress analysis	The technique to analyze the impact of stress on the voice output.
VSSR	Vibration space shift rate	The widest vibration space of the voice during a baseline (standard vibration space) compared with that encountered during a target situation.

Therefore, the aim of the current review is to carry out a literature search on patterns in voice stress that may offer insight into the potential psychophysiological mechanisms underlying the related voice-stress response. To meet this goal we will first frame stress and psychophysiological stress responses, explain the stages in voice production, then highlight the anatomical connections between the voice production system and the autonomic nervous system (ANS) and define the stressors that will be included for this review. The review outcome will comprise an overview of VSA parameters and present a framework of recurring patterns of voice parameters identified over studies that may point to underlying psychophysiological mechanisms important in stress regulation.

Stress has been conceptualized by Hans Selye as “the non-specific response of the body to any demand” (Selye, 1974, p. 14). Related to human performance, these demands can be physical and/or mental, evoked by internal and/or external environmental circumstances (e.g., noise, heat, cold, altitude, isolation…) (Moore and Trancoso, 1995; Hancock and Szalma, 2008). During coping with stress, an individual appraises his stressor and will experience a bidirectional exchange between processes of mental taxation and physiological reactivity (e.g., Lazarus and Folkman, 1984; Lazarus, 1991). So, a stress response does not occur solely within an individual nor within the environment, but it is a transaction between the individual and the environmental stress factor. This means that, in stress research, a unit of analysis cannot be restricted to either the subject or the stress factor itself, but that it should be evaluated as this reciprocal transaction (Hancock and Szalma, 2008). In a stress response, the voice occupies a more central role than one would expect at first sight. Both on long-term (Dietrich et al., 2008; Holmqvist et al., 2013) as in acute occasions (Giddens et al., 2013), stress can mark the voice output negatively. Maybe the most recognizable interrelation between the voice, stress and human physiology is stage-fright that can ruin a performance by physical voice and breathing impediments (e.g., Brantigan et al., 1982; Dietrich and Abbott, 2012). Other stressful professions that demand a large stress regulation, and in which the voice occupies a prominent position include those that rely on radio contact such as aviation operators and ground controllers (e.g., Simonov and Frolov, 1977; Simonov et al., 1980).

For the search into shared underlying psychophysiological mechanisms between the voice, the CNS and ANS during a stress response, the neurovisceral models (e.g., Thayer and Lane, 2000, 2009) and central autonomic network (Benarroch, 1993) offer valuable insights. From this perspective, a state of sympathetic arousal -and in extremis a life-threatening emergency- is considered as an alarm situation that is detected on a subcortical implicit level of awareness, demanding an explicit top-down modulation (LeDoux, 1996; Lane, 2008). In alarm detection, the amygdala and its connections to the thalamus, hypothalamus and pituitary, basal ganglia and brainstem nuclei are key role players (LeDoux, 1996). To prepare and organize an appropriate regulatory top-down response, the amygdala is bidirectionally connected with the components of the anterior cingulate cortex (ACC), prefrontal cortex and insula. The integrated output of this cortico-subcortical network is pivotal in the autonomic innervation of the sinoatrial node of the heart via the stellate ganglia and the NV (i.e., cranial nerve X). That is, the more top-down control of the central network, the more stimulation of the inhibitory action of the parasympathetic nervous system modulated by the NV (e.g., LeDoux, 1996; Lane, 2008). For instance, medial prefrontal cortex activity is known to be positively correlated with parasympathetic vagal inhibition in terms of heart rate variability (HRV) (Lane et al., 1998) and negatively correlated with heart rate (HR) (Drevets, 1999). Though, in a state of emergency that is associated with high arousal and reduced vagal tone, prefrontal explicit top-down mechanisms shut down and more automatic implicit processes take over (Lane, 2008).

To understand the impact of stress on voice production, it is necessary to take notice of the different steps in voice output. Voice production is the coordination of three processes, i.e., breathing, phonation and resonance (Kreiman and Sidtis, 2011). The first process, *breathing* is an automatically controlled process that serves the ultimate vital function of the exchange of gasses and thermoregulatory processes (Kreiman and Sidtis, 2011). During speech, we control the breathing (MacLarnon and Hewitt, 1999) by shortening inspiration and lengthening expiration (Estenne et al., 1990). When we speak, both respiratory (i.e., thorax and abdominal diaphragm) and laryngeal muscles are controlled by special efferent fibers of the NV. These fibers divide into the pharyngeal, superior laryngeal and recurrent laryngeal nerves (Câmara and Griessenauer, 2015). The recurrent laryngeal nerves further carry the motor signal of the special efferent fibers through the jugular foramen to the adductor and abductor of the intrinsic laryngeal muscles of the speech apparatus (Hermanowicz, 2007; Câmara and Griessenauer, 2015). For the second process to occur, *phonation*, the vocal folds must close and open again to create vibration. The frequency rate of these pulses determines the fundamental frequency (F0) of the vocal source contributing to the perceived pitch of the sound. To allow this vibration, phonation requires a balance between subglottal pressure (i.e., the pressure of the airflow below the glottis or space between the vocal folds) and the tension, stiffness, vocal fold approximation and resistance of the vocal folds (for an extensive explanation on the connectedness of each of these vocal fold processes and the respective muscles, see Zhang, 2015); a balance that is in general obtained by means of respiratory regulation with the aid of the abdominal muscles throughout the expiratory phase of speech breathing (Hixon et al., 1976) and laryngeal regulation by vocal fold adduction (Herbst et al., 2015; Zhang, 2015). Within this process, subglottal pressure determines vocal loudness whereas the adduction of the cricothyroid muscle impacts the F0 of the vocal source (Herbst et al., 2015; Zhang, 2015; Yao et al., 2016; Sundberg, 2017). When a balance is achieved, a relatively small glottal opening and low airflow that nevertheless is able to overcome the resistance, can be preserved. The airflow typically has to be maintained within a range of 0.15–0.5 L.s^-1^. So, an individual in a resting state, to start talking, needs an increase of expiratory airflow to maintain the minimal airflow required for phonation resulting in a ventilation rise of about 25% compared with a resting non-talking state (Bunn and Mead, 1971). However, when an individual is in a physical or mental state that already requires increased amounts of ventilation of the body beyond the maximal border of 0.5 L.s^-1^, ventilation needs to be downregulated during talking to maintain airflow compatible with clear speech. So, the breathing is the engine and the vocal fold vibration is the source of the sound that makes phonation occur. A third process, *resonance*, is offered by the oral cavities or containers of the vocal tract, i.e., the throat, mouth and nose in nasalized sounds (e.g., Clark and Yallop, 1990; Kreiman and Sidtis, 2011). In general physics, a resonator can be considered as a system that filters the vibrating source. It will pass the frequencies that are close to its own natural frequency and attenuate those that are further (Kreiman and Sidtis, 2011). The vocal tract encompasses multiple containers of air that vibrate at specific pitches entering by vocal fold vibration. Their resonant frequencies change by altering the shape and formation of the mouth, throat, lips etc. (e.g., Story et al., 1998). Consequentially, each vowel during speech has its own characteristic formant frequencies determined by the positions of the articulators (e.g., Clark and Yallop, 1990; Story et al., 1998; Kreiman and Sidtis, 2011) (referred to in literature as the formants or F1, F2, F3 etc.). Finally, to obtain speech, the articulatory speech organ needs to coordinate with these three processes involved in voice production. Taken into account the complexity of this coordination, stress can have an impact on each stage of voice production. Hence, the spatial change of the articulatory organs (lips, tongue parts, epiglottis, and larynx) or the transition from consonants to vowels is a demanding process required for fluent articulation (Kiss et al., 2014).

The output of both voice and stress responses rely on similar cardiorespiratory processes of the ANS. As stated above, the parasympathetic vagal system crucial in stress regulation (e.g., Berntson et al., 1991; Thayer and Lane, 2000, 2009) is also involved in voice and speech coordination (e.g., Câmara and Griessenauer, 2015). Although it has not yet completely been clarified in what way the NV may be of influence on speech (Yoshida et al., 1992; Hammer et al., 2015) and thus its stress reactivity, the missing link between both may be found in breathing parameters. Within the perspective of neurovisceral modulation (Thayer and Lane, 2000, 2009), a growing number of studies reports respiration to be the most sensitive parameter to stress (e.g., Boiten, 1998; Wilhelm et al., 2001; Van Diest et al., 2006; Pattyn et al., 2010; Vlemincx et al., 2010, 2011). Moreover, these studies point to the importance of a flexible respiratory system (Vlemincx et al., 2011), similar as the required flexibility described with regard to HRV and stress (Thayer and Lane, 2000). An increase in total respiratory variability during emotional states are said to be linked with emotional instability whereas decreases would be linked with certain processes of cognitive load that involve top-down regulation (Vlemincx et al., 2010, 2011). Anatomically, the NV may thus interconnect the stress responses in both, stress, breathing and speech by means of its nerve branches (e.g., Yoshida et al., 1992; Câmara and Griessenauer, 2015; Hammer et al., 2015). For instance, the superior laryngeal nerve innervates the cricothyroid muscle that is involved in vocal fold stretching and, thus, pitch regulation (Kreiman and Sidtis, 2011) and the recurrent laryngeal nerve controls all of the other intrinsic laryngeal muscles. The fibers that form the superior laryngeal nerve split from the NV travel through the carotid artery to innervate these laryngeal muscles (Câmara and Griessenauer, 2015).

Hence, knowing that voice production is clearly integrated within the ANS (Câmara and Griessenauer, 2015) and that the ANS does not respond independently from the CNS in a human stress response (Thayer and Lane, 2000, 2009) in the current review we would like to shed light on VSA by approaching it from this neurovisceral integrated perspective. Based on the taxonomy of stressors established at the ESCA-NATO workshop on ‘Speech under Stress’ in Lisbon (Moore and Trancoso, 1995), we categorized stressor types in five classes, i.e., physical load; alcohol/sleep-deprivation/hypoxia; emotional load; cognitive load and the combination of the two latter.

## Methods

Studies were identified via literature searches in the Web of Science, Scopus and the PsycInfo databases using the keywords “voice/speech/speaking” in combination with “stress,” “stress analysis,” “physical,” “alcohol,” “sleep,” “hypoxia,” “flight,” “emotions,” “cognitive,” “human performance.” Further, we did additional searches based on the references encountered in the consulted studies. We took into account all the articles published until 01/02/2018. We only included those studies in which VSA was based on a real acoustical analysis and not a subjective observation. We did not include studies on voice recognition. Voice recognition research aims to find methods to filter stress confounds rather than to highlight them in order to optimize automatized speech recognition (e.g., Hansen and Patil, 2007; Brumm and Zollinger, 2011). We also did not include studies on lie-detection since this research field has been carefully reviewed by Giddens et al. (2013).

## Results

Here we will first review the speech parameters we encountered related to their respective stage in the process of speech production (i.e., breathing, phonation and resonance), the stressors that impact them and their anatomical significance (see also Table 2). Subsequently, the effects on voice output of the different types of load will be reviewed. With regard to the speech parameters, we only included those variables for which we found at least a brief explanation, methodological background and/or rationale. Variables that were not explained in the method section were not included in our results. Finally, we want to mention that we reported—in accordance with the reviewed papers—absolute F0-values and ranges in Hz and not the logarithmical values.

**Table 2:** Overview of the speech variables related to their respective stage in the process of speech production (i.e., breathing, phonation and resonance), the stressors that impact them.

Speech parameters	Speech process	Stressors impact	Remarks
• Respiration rate	Breathing	Physical load	Competition between ventilation processes speech
• Articulation rate			and metabolic demands muscles: inappropriate breathing
• Word duration			pause placements.
• Vowel duration			
• Respiration time between words or sentences			
• Voice Onset Time (VOT)			
• Voicing and voiceless transients			
		Acute hypoxia	Different impact on speech between chronic and acute hypoxia.
		Alcohol	Slurred speech.
		Emergencies	Faster articulation rate.
**F0-based variables:**			
• Mean F0 SD	Phonation	Physical activity	Fatigue vs. metabolic response?
• Min to max range			
• F0 peaks			
• F0 floor values			
• Relative average perturbation			
		Acute hypoxia	Difference between chronic and acute hypoxia.
		Alcohol	Replication study needed.
		Sleep deprivation	Impact in correspondence with circadian rhythm.
		Emergencies	Real-life stress clear impact but influence of voluntary control.
		Cognitive workload	Challenge to differentiate between emotional and cognitive load.
		Different types of emotions	Variable results.
• Jitter	Phonation	Emergencies	Decrease jitter: only one study with *N* = 1.
• Shimmer			
		Cognitive workload	Decrease jitter Decrease shimmer.
• Signal to noise ratio (SNR)	Phonation	Alcohol	Strong indicator in combination with F0.
• Harmonic to noise ratio (HNR)			
Harmonic Richness Factor	Phonation	Physical activity	Subject dependent.
Harmonics	Phonation	Different emotions: anger, neutral with little sadness and loudness	Only one study found.
Formants	Resonance	Physical load	Only one study with lot of non-responders.
		Emotional load – Emergency	Significant variations between stress and non-stress but not for all the types of vowels, with different senses of variation on vowel type with stress arousal.
		Cognitive load	F1, F2, and F3 are vowel specific. F1/F2 ratio potential to differentiate between low and high cognitive load.
Glottal flow	Resonance	Physical load	Increased open and closing quotient indicative of a breathy voice – decreased open and closing quotient of a pressed voice.
NAQ	Resonance	Physical load	Potential for NAQ – F0 combination.
MFCC	Resonance	Sleep deprivation	Circadian pattern
		Emotional load	Vowel-dependent? Important to preselect appropriate mel-filters.

### Speech Parameters

#### Breathing

With regard to the stage of breathing, a series of duration-related variables are of interest, representing stress-induced respiratory perturbations directly and/or indirectly impact voice production. For instance, an increased or irregular respiration rate obviously leads to shorter speech periods between breaths along with inappropriate respiration places (e.g., Baker et al., 2008). This affects the temporal pattern and articulation rate of speech (Hansen and Patil, 2007). The number of syllables per phrase (i.e., between the inspirations) (SPP), inappropriate breathing pauses (IP) and the articulation rate (AR) (i.e., SPP divided by the total length of the sample minus the duration of pauses) are often used duration variables. As such, the efficiency of control and coordination of laryngeal-oral movements can be derived from the time intervals between vowels and consonants. For instance, Voice Onset Time (VOT), the time interval between the release of a plosive such as ‘p,’ ‘b’ and the beginning of the vocal fold vibration associated with the subsequent vowel gives information about the coordination of the articulatory apparatus. Related to VOT, some authors make use of the delta measures of voicing (e.g., ‘b’) and voiceless (e.g., ‘p’) transients.

#### Phonation

During phonation, stress increases the tension of the cricothyroid muscle and intensifies subglottal pressure (Zhou et al., 2001). As mentioned above, increased tension of the cricothyroid muscle impacts the frequency of vocal fold vibration (Kreiman and Sidtis, 2011; Zhang, 2015) whereas increased subglottal pressure has an impact on the vocal loudness (Herbst et al., 2015; Zhang, 2015; Yao et al., 2016; Sundberg, 2017). As said, periodic vocal fold vibration is measured by the fundamental frequency or so called F0 of a sound which directly expresses the number of cycles per second (Hz) of a sound wave. Within VSA, F0-variables provide useful information about the ongoing processes in the laryngeal nerves and cricothyroid muscle system. Common measures are the variation of a speaker’s average F0, F0 SD, the minimum–maximum range and the most extreme F0-peaks. We also encountered F0 floor values and relative average perturbation as indexes for voice stress. F0 floor values refer to the lowest F0 values documented by taking the frequency below which the lowest 5% of F0-values are located. Relative average perturbation (RAP) is the mean difference between consecutive cycles in the F0, divided by the mean period.

Independent from the frequency of vocal fold vibration, each sound produced by the voice comprises its typical signature, i.e., the timbre, quality or how a voice sounds. This timbre is the result of the combined output of the relative strength of the different subcomponents or harmonics of the sound that can be obtained by spectral analysis. A measure that gives insight into the general harmonic richness of a sound or voice quality is the harmonic richness factor (HRF) which refers to the ratio of the sum of the amplitudes of the harmonics and the amplitude of the component at the fundamental frequency (Godin and Hansen, 2015). However, voice quality is not just a question of the amplitude mapping of the harmonics. It is also defined by the fact that a human voice is never perfectly periodic. A voice without a certain irregularity or perturbation would sound very mechanical or computerized (Murphy, 2000). Small short-term aperiodicity in a speaker’s phonation in the period length and amplitude occur from cycle to cycle (Clark and Yallop, 1990). In a former study (Kuroda et al., 1976), voice inconsistencies were measured as the mean vibration space of a voice (i.e., the space between the vertical deflections of the sound spectrogram) or the vibration space shift rate (VSSR), calculated by comparing the widest vibration space of the voice during a baseline (standard vibration space) with that encountered during an emergency situation. Other techniques for voice inconsistencies or noise in the voice are jitter (i.e., short-term changes in period length) and shimmer (i.e., short-term changes in amplitude) (e.g., Clark and Yallop, 1990; Dietrich and Abbott, 2012; Boersma and Weenink, 2013). When related to stress, jitter and shimmer are the result of either small variations or asymmetries in the cricothyroid muscle tension (Brenner and Shipp, 1988) and/or fluctuations in subglottal pressure (Yao et al., 2016) and/or perturbations in the mucous of the vocal folds (Higgins and Saxman, 1989; Kreiman and Sidtis, 2011). Both for jitter and shimmer, there are standard norm values and a threshold of pathology based on the ratios of averaged differences in rate (jitter) or amplitude (shimmer) of consecutive periods divided by the average rate or amplitude (Boersma and Weenink, 2013). These thresholds are set on 1.040% for jitter and 3.810% for shimmer. Moreover, jitter and shimmer have an additional value to the information derived from F0 fluctuations because they occur relatively independent from prosodic patterns (Rothkrantz et al., 2004). This prosody-independent feature of shimmer and jitter variables has benefits in noisy contexts (e.g., a flight cockpit) (Gopalan, 2000). Furthermore, signal to noise (SNR) and the harmonic to noise ratio (HNR) (e.g., Patel et al., 2011) indicate the amount of periodicity against aperiodicity in the voice. High HNR or SNR values refer to a clear voice with high periodicity. The opposite is the case for pathological, breaky or whispering voices.

#### Resonance

As explained, by changing the form and the size of oral cavities and with that the resonance frequencies of them, we can amplify and filter certain frequencies within a phonation signal. The set of most salient frequencies of the most pronounced resonating oral cavities of each vowel are the formants of a sound (Ladefoged, 1967; Clark and Yallop, 1990). It is common to report the three first formants, F1, F2, and F3 (e.g., Clark and Yallop, 1990; Kreiman and Sidtis, 2011; Boersma and Weenink, 2013). It is suggested that F3 is important in the identification of different lip positions in vowels with a similar height and fronting position (Ladefoged, 1967). The pattern of the most salient formant peaks or amplified frequencies (e.g., Titze and Scherer, 1983) are sometimes called “a vocal tract transfer function” (Titze and Scherer, 1983). It is a continuous curve describing the ratio between the acoustic input and output for the vocal tract which refers to how the vocal tract transfers source energy of the vocal fold vibration to its final acoustic output. Since formants give insight into the functioning of the vocal tract and the laryngeal muscle system, they are also of interest for articulatory perturbations. Any change in the control of the laryngeal and/or pharyngeal system may be reflected in the use of the oral cavities (Kreiman and Sidtis, 2011; Hansen and Patil, 2007).

Some measures are based on a technique of inverse filtering to capture the activity of the glottal voice source waveform, the sound produced by the pulsating transglottal airflow. The aim of this procedure is to remove the effects of natural vocal tract filtering and to obtain information regarding the quality of phonation (e.g., Alku et al., 2002) or vice versa to gather the vocal tract signal (Cummings and Clements, 1992) by measuring the duration and instant of glottal closure (e.g., Hansen, 1989; Godin and Hansen, 2015). The glottal waveform of a vowel, for instance, is a cycle of closed and open phases (Godin and Hansen, 2015). Based on the nature of this cycle, the timing of open and closing quotients (OCQ) can be indicative of stress (Godin and Hansen, 2011). Concretely, an increased OCQ is indicative of a breathy voice whereas a decreased OCQ is related to a ‘pressed’ voice (Alku and Vilkman, 1996). Another measure for ‘breathiness’ of the voice is the normalized amplitude quotient (NAQ) (Alku and Vilkman, 1996; Alku et al., 2002; Campbell and Mokhtari, 2003; Godin and Hansen, 2015). NAQ is the ratio of the maximum peak-to-peak amplitude of the glottal flow to the minimum of the glottal flow derivative, normalized by the fundamental period and the sampling frequency (Alku et al., 2002; Godin and Hansen, 2015). In comparison with neutral speech, an increased NAQ indicates a breathy phonation, whereas a decreased NAQ is the result of a pressed phonation (Alku et al., 2002; Godin and Hansen, 2015). Within a sound cycle, the dominant frequency components corresponding to the energy peaks of the spectrum can be further finetuned by means of a cepstral analysis. A cepstral analysis comprehends the calculation of a discrete number of coefficients called Mel-frequency cepstral coefficients (MFCCs) according to the equally spaced Mel-scale to approximate the critical bands of the human ear (Rabiner and Juang, 1993; Molau et al., 2001). However, other inverse infiltering methods can be used (see for instance Alku et al., 2002 for more detailed information on inverse filtering methods and Arroabarren and Carlosena, 2003 on the strengths and limits of this method). Once the Mel-filters are selected, the Mel-spectrum can be obtained. So, MFCCs can be considered as a spectrum of a spectrum (Kreiman and Sidtis, 2011) and are indicative of breathiness in the voice (e.g., Hillenbrand and Houde, 1996).

### Effect of Stressors

#### Physical Load

The impact of physical load on speech is argued to be due to an internal competition that occurs in the body between the ventilation processes required to speak and those to meet the metabolic demands of the exercised muscles. Speech already puts some constraints on the range of expiratory airflow (e.g., Baker et al., 2008) inducing consequently spontaneous respiratory variability (e.g., Tininenko et al., 2012). When combining exercise with speech, the most salient and most reported expression of this respiratory competition in speech is the occurrence of linguistic inappropriate breathing pause placements (e.g., Otis and Clark, 1968; Bunn and Mead, 1971; Phillipson et al., 1978; Doust and Patrick, 1981; Meckel et al., 2002; Baker et al., 2008; Rodríguez-Marroyo et al., 2013).

We found six studies that examined the impact of physical load on the voice (see Table 3). In a former study, Mohler (1982) reported indications for a linear relationship between F0 and physical load in terms of dyspnea, oxygen consumption (VO_2_) and ventilation. Mohler (1982) studied 44 healthy male subjects that were exercised to their maximal oxygen consumption (VO_2_max) by an incremental treadmill test with 4 min exercise intervals (or until the subject was tired) and 15 min rest periods in between. A speech sample (i.e., an elongated ‘a’ for 3–5 s) was recorded at each third minute of the exercise and the anxiety state of the subjects was included as a variable. However, the linear physical load/F0 relationship did not persist. Firstly, linearity was not found when physical workload was low and secondly there was an influence of the participants’ anxiety state as such that elevated starting F0 values in anxious subjects caused ceiling effects. Johannes et al. (2007) further examined the existence of this linear relationship in a study on 11 male members of the Austrian Special Forces elite unit. The participants underwent a standard cycle test with progressively increased workload (every 2 min increase by 25 W) until a breaking point of exhaustion was reached. Every 30 s before increasing the physical load and 1, 3, and 5 min after physical exhaustion, the subjects had to count to 10. The interaction between physical load and speech proceeded in multiple plateaus rather than in a linear format. Although significant increases in HR and systolic blood pressure (BP) were observed in relation with increasing physical workload, there was no increase in F0 as long as the physical load was well tolerated by the subjects (i.e., workload between 100 and 200 W). It was only at the pre-exhaustive stage and at the breakpoint of submaximal and maximal effort that F0 showed significant differences in comparison with the rest-level and tolerated level (Johannes et al., 2007).

**Table 3:** Studies on the impact of physical load on voice and speech production.

Study	Speech process	Subjects	Context	Task	Speech measures	Results
Baker et al., 2008	Breathing	*N* = 12 (6 males)	Laboratory	Aerobic task with progressive workload at 50% and 75% of VO_2max_: speaking and no-speaking condition. Baseline with six additional time points at 50% of VO_2_max and two at the 75% of VO_2_max. Speech task: 15 s standardized novel fragment every 3 min.	SPP AR IP	• SPP decreased in the 50 and 75% of VO_2_max speaking tasks.
						• IP increased the 50 and 75% of VO_2_max speaking tasks.
						• AR no change.
Godin and Hansen, 2008	Phonation Resonance	*N* = 51 (9 males)	Laboratory	35 standard speech sentences. Physical activity on an elliptical stair stepper.	F0 F0 SD Utterance duration Voiced – non voiced frames Formants	• Speaker independent correlates: percentage of voicing (decrease in 88.2% of the participants).
						• Speaker dependent correlates: F0 (increase in 60.8%, decrease in 13.6% and no change in 25.5% of the participants), F0 SD (no significant impact), utterance duration (50–50%), glottal waveform and formant parameters (significant shift in F1 but many non-responders).
Godin and Hansen, 2011	Phonation Resonance	*N* = 4 (2 males)	Laboratory	Five repeated series of eight vowel-consonant-vowel (VCV), and eight consonant-vowel (CV) utterances in BL (seated) and during physical load.	F1, F2 OQ	• F1: interaction effect between speaker and physical load.
						• F2: main effect of physical load.
						• OQ: interaction between speaker, vowel, and physical load.
Godin and Hansen, 2015	Phonation Resonance	*N* = 78 (gender counterbalanced)	Laboratory	65 readings of 15 s. (Non)native read and spontaneous speech. Maintaining 10 mph on an elliptical stair stepper.	NAQ HRF F0	Correlation between F0, NAQ, and HRF shift: a shift in F0 on the entire sample showed significant correlations with a NAQ shift (*r* = 0.53) and HRF shift (*r* = -0.34) and there was a strong correlation between NAQ and HRF (*r* = -0.89).
Johannes et al., 2007	Phonation	*N* = 11 (male)	Laboratory	Standard cycle test progressively increased load until breaking point of exhaustion. Speech task: counting 1–10.	F0	Increased F0, only at submaximal and maximal effort.
Mohler, 1982	Breathing Phonation	*N* = 44 (male)	Laboratory	Incremental treadmill test with 4 min exercise – 15 min pause intervals. Speech test: 3–5 s single ‘a.’	F0	Linear relationship between F0 and physical load in terms of dyspnea, oxygen consumption (VO_2_) and ventilation (VE). Anxiety creates ceiling effect (i.e., higher F0 onset in anxious state).

In a study by Baker et al. (2008), 12 healthy male participants passed through a standard graded exercise test protocol on a stationary cycle ergometer that consisted of a non-exercise baseline and progressive workload at 50 and 75% of VO_2_max. Besides VO_2_max, ventilation and HR, SPP, IP, and AR were measured. SPP significantly decreased over time in both the 50 and 75% of VO_2_max speaking tasks. However, AR did not, which implies that individuals took more inspirations during increased exercise. IPs significantly increased over time in both the 50 and 75% measures.

Godin and Hansen (2008) examined speech in 51 participants (9 males) during physical load in comparison with neutral speech and concluded that there are speaker dependent and independent correlates. This study was based on the UT-Scope corpus for speech under cognitive and physical load (Varadarajan et al., 2006). All of the participants had to execute the same task which resulted in different levels of exertion for each subject. The task required participants to maintain a 10 mph speed on an elliptical stair stepper in a protocol of alternating physical activity and speech standard sentences during 16 min 15 s. Breaks were allowed but there were no details regarding breaks or exertion levels reported. F0 was considered a speaker dependent variable. There was no significant impact on the F0 SD. Changes in duration of the utterances were speaker-dependent with as many observed increases as decreases. Voiced speech was considered speaker-independent and formant location shifts were considered speaker-dependent. In 2011, Godin and Hansen showed that physical load affects F1, F2, and the OCQ in vowel production. However, F1 was speaker dependent and OCQ was speaker and vowel dependent. In 2015, Godin and Hansen studied the impact of physical load on voice quality in terms of NAQ, HRF and F0 on 78 participants with varying fitness levels in an elliptical stair stepper task and standardized speech test. The impact of physical workload was dependent from both task type and speaker. There was a large interpersonal variability with a pattern of responders and non-responders resulting in small overall changes. However, the patterns of combined parameters, rather than a solely individual parameter were indicative (see Table 3).

#### Deleterious Impacts on Human Performance: Alcohol, Sleep Deprivation and Hypoxia

##### Alcohol

We found five studies on the impact of alcohol on voice and speech output (see Table 4). Alcohol has been documented to induce slurred speech, i.e., slowed down, dysfluent speech with more interjections, omissions, errors, perturbated suffixes, poorer articulation (e.g., Trojan and Kryspin-Exner, 1968; Sobell and Sobell, 1972; Sobell et al., 1982). With regard to phonation, Sobell et al. (1982) found no significant effect on F0, neither in moderate nor high alcohol doses. However, a study by Klingholz et al. (1988) on 11 male subjects suggested that it is not F0 itself that should be analyzed, but rather its variance. Klingholz et al. (1988) showed significantly increased variance ranges between peaks and valleys due to alcohol intoxication. Moreover, the combination of SNR and the F0 parameters offered a robust indicator with a near to perfect detection rate even in lower intoxication whereas the F1/F2 ratio became only sensitive in high levels of intoxication with interindividual differences (Klingholz et al., 1988). In another study (Brenner and Cash, 1991), the speech samples of the captain engaged in the Exon Valdez oil disaster (who was witnessed to be intoxicated) before and after the disaster were analyzed. The samples 1 h before and after the accident showed speech deteriorations typical for intoxication such as significantly less syllables per seconds and more errors in comparison with 33 h before the accident.

**Table 4:** Deleterious impacts on human performance: studies on the impact of alcohol, sleep deprivation and hypoxia on voice and speech production.

Study	Speech process	Subjects	Context	Task	Speech measures	Results
**Alcohol**					
Brenner and Cash (1991)	Phonation	*N* = 1	Real-life	No task. Speech samples of the captain engaged in the Exxon Valdez oil disaster 33 h before, 1 h before and 1 h after the disaster.	Speech rate, articulatory errors	• Less syllables per hour.
						• Increased speech errors.
Klingholz et al., 1988	Phonation Resonation	*N* = 11 (male)	Laboratory	Reading task: text in sober and intoxicated condition.	F0 and SNR F1/F2 ratio	• Combination F0 and SNR robust detector, 0% error rate; F0 2.8%; SNR 3.2%.
						• The F1/F2 ratio responded only in high intoxication.
Sobell and Sobell, 1972	Breathing-phonation	*N* = 16 (male) – alcoholic subjects	Laboratory	Three separate reading sessions: sober, mild, moderate intoxication with interval of 48 h. Reading task: linguistic passage of 613 words.	Reading time, interjections, omissions	• Increased mean reading time.
						• Increased interjections.
						• Increased omissions.
Sobell et al., 1982	Phonation	*N* = 16 (male) – non-alcoholic moderate to heavily social drinkers	Laboratory	Three separate reading sessions: sober, moderate, high intoxication.	Reading rate Amplitude in dB F0	• Decreased reading rate.
						• Decreased amplitude (from sober to moderate).
						• No impact on F0.
Trojan and Kryspin-Exner, 1968	Phonation	*N* = 16 (male)	Laboratory	Reading task.	F0 Articulation rate	• F0 mixed results.
						• Decreased articulation rate.
**Sleep deprivation**					
Greeley et al., 2006	Resonance	*N* = 6 (no information on gender)	Laboratory	List of 31 words read at six time points (10:00 AM, 4:00 PM, 10:00 PM, 4:00 AM, 10:00 AM, and 4:00 PM) through a 34 h sleep deprivation period.	SAFTE sleep reports MFCC: 12000 formant frequencies	• Correlation between fatigue score and Mel-frequency cepstral coefficients (MFCCs).
						• Circadian periodicity in both sleep and voice measures.
						• Character “p” in particular sensitive.
Whitmore and Fisher, 1996	Phonation	*N* = 12 (male)	Laboratory	• Sleep deprivation 36 h, some naps allowed.	FO Word duration	Circadian pattern in cognitive performance and voice aspects: during early A.M. hours lowest cognitive performance – increased F0 – decreased word duration.
				• Speech semi-standard sentence including standard words (e.g., ‘Futility Magelan’).		
				• Speech non-standard words (e.g., the pilot’s name and zulu-time).		
				• Cognitive matrix comparison task, a logical reasoning task, a tracking task, attention switching task and a recognition task.		
**Hypoxia**					
Kiss et al., 2014	Breathing Phonation	Chronic hypoxia *N* = 9 (no information on gender)	Real-life	Short text phonetically balanced folk tale (about six sentences North Wind and the Sun”).	Articulation rate Transient segment rate	• Articulation rate: no effect.
						• Transient segment rate: decrease and similar shape but with a 30 days shift delay with regard to SO_2_-dip.
Lieberman et al., 2005	Breathing Phonation	Chronic hypoxia *N* = 36 (no information on gender)	Real-life	Cognitive test battery for Parkinson Disease patients: sorting card tests – Wisconsin Card Sorting Test, the ‘Odd-Man-Out test’ (OMO test), Mini-Cog Quick Assessment Battery of Shephard and Kosslyn (2005) Reading task: 30 monosyllabic English words with voiced and non-voiced syllables.	VOT Vowel duration Comprehension errors.	• Decreased VOT separation time.
						• Increased vowel duration.
						• Increased comprehension errors.
Saito et al., 1980	Breathing Phonation	Acute hypoxia *N* = 1 (male)	Real-life	No task. Fatal air crash.	F0 VOT	• Decreased F0.
						• increased VOT.

##### Sleep deprivation

We found two studies that examined the impact of sleep deprivation on voice production. Both studies described the existence of a circadian trend (see Table 4). Whitmore and Fisher (1996) analyzed F0 and word duration during a bomber simulation mission task of three 36-h experimental periods (during which the participants were sleep deprived) with an inter-period of 36-h of recovery in 12 qualified bomber aircrew members. During sleep deprivation, the participants were allowed to take some voluntary short naps. However, no information was provided on the number, duration and moments that these naps were taken. Speech data, cognitive test data and subjective fatigue data were collected approximately every 3 h during each mission. Almost all of the dependent variables (i.e., subjective rated fatigue reports, voice aspects and cognitive performances) reflected a circadian pattern. Cognitive performance was lowest during early A.M. hours which was reported to be mirrored in the voice performance. Indeed, both F0 and word duration were significantly decreased on approximately the same early A.M. hours. Also Greeley et al. (2006) found a circadian pattern in MFCCs due to sleep deprivation. Voice perturbations were compared with sleep measures of the Sleep, Activity, Fatigue, and Task Effectiveness (SAFTE) reports (Hursh et al., 2004). Six participants were asked to recite a list of 31 words at six time points matching the human circadian rhythms. Correlations were searched between sleep onset latency (i.e., the time that it takes for a person to fall asleep) determined by an electroencephalography (EEG) and MFCC voice parameters. A strong correlation (*r* = -0.89) for the phoneme ‘p’ and for ‘t’ (*r* = -0.67) was found (Greeley et al., 2006). In both studies, it has been concluded that voice acoustics are promising in the detection of sleep-deprivation.

##### Hypoxia

We found three studies on hypoxia, two on chronic and one on acute hypoxia (see Table 4). Hypoxia (i.e., insufficient oxygen supply) deprives the body of its full capacities to meet crucial metabolic needs which results in psychomotor, physiological and cognitive deterioration (Petrassi et al., 2012). Chronic hypoxia comes about during long-term exposure to, e.g., mountain altitudes whereas acute hypoxia is present in an aviation context. The danger of acute hypoxia is hidden in the insidiousness of the phenomenon. From the moment pilots may notice the first signs of hypoxia, their cognitive and psychomotor functioning may already be deteriorated (Petrassi et al., 2012). In a study of Saito et al. (1980), the analysis of pilot communication just before a fatal crash was compared with that of a subject that read aloud the same words in a hypobaric chamber. Although normally the emotional stress of an emergency situation causes dramatic F0 increases (see further, emotional load), Saito et al. (1980) found in both voice fragments a decreased F0, even just before the crash, suggesting to be consequential of another process impacting the voice. Besides decreased F0, Saito et al. (1980) observed increased VOT and blurred formant frequencies.

The impact of chronic hypoxia on voice duration parameters has been investigated in a study at the Concordia Station in Antarctica located at 3233 m altitude on a crew of 9 persons over a stay of 150 days (Kiss et al., 2014). Kiss et al. (2014) found no impact on AR, but they found a decrease in transient segment rate. This decrease ran in parallel with the SO_2_-course, however, with a 30 days shift delay. After approximately day 80, a recovery was seen (Kiss et al., 2014). In another study (Lieberman et al., 2005), both speech and cognitive performance were measured before, during and after a 48 h climbing journey to Mount Everest (8848 m). In the speech task, the participants were asked to read aloud a list of 30 monosyllabic English words starting and ending with stop consonants, such as “bat,” “goat,” and “dad.” Lieberman et al. (2005) found a decreased VOT separation time and increased vowel duration related to increased cognitive comprehension errors.

#### Emotional Load

Real-life emergency situations are suggested to evoke acute stress responses to facilitate remedial action (Scherer, 2005; Kreibig, 2010). Firstly, we want to report six studies on VSA of radio communications during a life-threatening emergency situation and one study during a non-life-threatening emergency (see Table 5). In general, these studies reported consistently increased F0 in response to emergency stress. In a study by Mayer et al. (1994) that analyzed radio pilot communication during a routine check versus emergency prior to a helicopter crash, little change was measured in speaking rate. However, syllable count significantly decreased, F0 increased from 123.9 to 200.1 Hz and the range from 124.2 to 297.3 Hz. Similar large F0 increases were observed during a flameout emergency situation with a F-16 pilot and other flight emergencies (Brenner et al., 1985). With regard to voice inconsistencies, Kuroda et al. (1976) pooled and analyzed the voice communications in terms of VSSR of pilots involved in 14 actual aircraft accidents, eight of them fatal. Although individual reactivity patterns were present, a relationship was reported between the stress-level and VSSR. High VSSR-levels at the initial stage of an emergency situation were characteristic in fatal or highly critical flights (Kuroda et al., 1976). Finally Brenner et al. (1985) found decreased jitter measures during emergency states.

**Table 5:** Studies on the impact of emotional load on voice and speech production.

Study	Speech process	Subjects	Context	Task	Speech measures	Results
Benson, 1995	Phonation	*N* = 1 (pilot)	Real-life	No task. Crash vs. routine check.	F0	Increased F0: 115–163 Hz
						Increased F1: 510–537 Hz.
Brenner et al., 1985	Phonation	*N* = 1 (pilot)	Real-life and laboratory	No task. Pilot communication.	F0	• Increased F0: 95–148 Hz; 101–123 Hz; 149–264 Hz.
					F0	• Increased F0 SD: 12.9–23.7 Hz; 12.6–63.8 Hz; 30.1–66.0 Hz.
					SD Jitter	• Decreased jitter: 1.90–1.53%.
Demenko and Jastrzêbska, 2012	Breathing Phonation	*N* = 45	Real-life	No task. Phone Emergency calls in function of different types of emotions.	F0	• Fear: increased F0, F0 range and speech rate with high maximal peak frequencies.
					F0 range speech rate (i.e., syllables per s) maximal peak frequencies	• Anger – irritation: increase F0 and F0 range.
Hansen and Patil, 2007	Breathing Phonation	400 F0 contours (number of participants not mentioned)	Real-life	Stress conditions from SUSAS corpus for anger.		• Increased F0, F0-variance and F0-range,
						• Increase in formants F1 and F2,
						• Increased vowel duration and increased word intensity.
Kuroda et al., 1976	Phonation	*N* = 14 (pilots)	Real-life	No task. Pilot communication of 14 aircraft accidents.	VSSR	A higher VSSR in the start of the emergency communication related with more critical/fatal accident.
Mayer et al., 1994	Phonation	*N* = 1 (pilot)	Real-life	No task. Crash vs. routine check.	Speech rate	• Speaking rate little impact.
					Syllable count	• Syllable count significantly decreased.
					F0	• Increased mean F0: 123.9–200.1 Hz
					F0-range	• Increased F0 range from 124.2 to 297.3 Hz.
Postma-Nilsenová et al., 2016	Phonation	*N* = 1 patient during a clinical interview	Real-life	No task. Interview.	Galvanic skin response Mean and SD of F0, RAP and jitter	• Negative correlation between GSR and F0 SD.
						• Increased jitter levels in emotional load speech fragments.
Ruiz et al., 1996	Phonation Resonance	*N* = 2 (pilot and co-pilot)	Real-life	No task. Three stress stages before crash: Stress 0 (neutral), Stress 1 (first incident), Stress 2 (final incident before a crash).	F0	• F0-increase during stress 1 in pilot (117–150 Hz) and only during stress 2 in co-pilot (to 204 Hz with a maximal frequency of 340 Hz).
					Formants	• Significant increase in F2 in the pilot and a significant decrease in F3 in the co-pilot.
Sigmund, 2006	Phonation Resonance	*N* = 31	Real-life	Exam stress – public presentation.	F0	• Increased F0 and V0 variance.
					F0 variance	• Increased F1 and F2 frequencies.
					MFCC	• Decreased mel-cepstral coefficients.
					Formants	
Streeter et al., 1983	Phonation	*N* = 2 (system operator and superior)	Real-life	No task. Radio communication during.	F0	• System operator small decrease in F0 (138–136 Hz), stable F0-range (22 Hz), decreased F0 max (202–197 Hz), decreased speech rate (4.6–4.1 words/s). Stable amplitude.
					Speech rate	Superior small increased F0 (147–155 Hz), and F0-range (20–26 Hz), increased F0 max (193–218 Hz) decreased speech rate (5.3–4.8 words/s), increased amplitude and decrease in speech rate.
					Amplitude.	
Williams and Stevens, 1972	Phonation	*N* = 1 (pilot)	Real-life	No task. 1968: Crash vs. routine check. 1972: radio reporter.	F0	Increase F0: 208–432 Hz.

Hence, an emergency situation appears to trigger spontaneous dramatic F0-increases in the voice. However, there are some reports that have shown that the impact on the voice can be regulated by an individual to a certain extent. For instance, in a study of Ruiz et al. (1996), a laboratory stress situation and real-life emergency situation were compared. Both stress situations were divided into three stress stages, i.e., normal neutral stage (stress-0), first stress induction or first incident (stress-1) and high stress induction or the final incident before a crash (stress-2). The emergency analyses were based on a conversation between a pilot and co-pilot. In the pilot, a large F0-increase was observed mainly in the stress 1 condition, whereas the co-pilot only showed a dramatic increase in the stress-2 condition. With regard to the formant structure, Ruiz et al. (1996) found a significant increase in F2 in the pilot and a significant decrease in F3 in the co-pilot. The different voice-responses observed in the pilot and co-pilot were interpreted as a potential result of the professional function and expected agency and coping (i.e., the co-pilot as a more mediating function). A similar difference in voice reactivity, probably related to diverse stress coping habits, was found in Streeter et al. (1983). They analyzed the communication calls between a system operator and his superior before and during the 1977 New-York blackout, a non-life-threatening situation. The descriptive statistics showed none to very small differences in the voice parameters of the system operator for F0, F0 SD, maximum F0 and speech rate. The changes in the superior were also small, however, F0-parameters were oriented in the other direction. That is, a small increase in F0, F0 SD, maximum F0 and decrease in speech rate. Also with regard to amplitude, the findings showed an increased average amplitude, SD and maximum amplitude in one caller whereas all of these aspects remained stable in the other.

Demenko and Jastrzêbska (2012) reported the analysis of a few hundred emergency police calls, selected from an initial database of 6,000 calls. In this study, emotional load was related to different types of emotions. Stress related to fear showed increased F0, F0-range and speech rate (i.e., syllables per s) with high maximal peak frequencies during anxiety. Also anger and irritation were characterized by an increase in F0 and F0-range in relation to neutral speech (Demenko and Jastrzêbska, 2012). Another study (Hansen and Patil, 2007) also showed increased F0 and F0-variance, an increase in formants F1 and F2, increased vowel duration and increased word intensity in angry samples.

Finally, two studies (Sigmund, 2006; Postma-Nilsenová et al., 2016) examined a multidimensional stress response during emotional load. In Postma-Nilsenová et al. (2016), the relationship between galvanic skin responses (GSR) as a physiological correlate of emotional load and speech acoustics (F0, F0 SD, RAP, and jitter) between a physician and a patient during a clinical interview were measured. For each skin conductance (SC) interval, the corresponding vowel fragment of the patient’s speech (V2) was extracted, as well as the 600 ms of speech immediately preceding (V1) and immediately following the interval (V3). Postma-Nilsenová et al. (2016) found a negative correlation between SC-levels and F0 SD, slope and increased jitter levels in V3. In Sigmund (2006), a Czech speech database to examine speech under exam stress was created. Speech and HR were measured during the exam and a few days after the exam as a baseline. HR-measures were meant to objectively control the actual stress levels during the exam. Under stress, HR increased, F0 and F0 SD increased, F1 and F2 increased and MFCCs decreased in relation to a baseline (Sigmund, 2006).

#### Cognitive Load

Cognitive load can be considered as the extra effort that needs to be generated to overcome a discrepancy between the environmental demands of a task and one’s level of resources (Grassmann et al., 2017). In total, we found five studies that examined a direct impact of cognitive load on voice output.

In Hecker et al. (1968), F0-patterns of five participants during task-induced cognitive load controlled by setting time-constraints in a read-aloud calculation task were studied. They found individual reactivity in F0. However, in a later study of Griffin and Williams (1987) a direct association between F0 and task complexity was observed. In this study, 20 participants were subjected to a series of psychomotor tests with increasing difficulty (see Table 6). Increased task complexity resulted in significantly increased F0, increased peak amplitude or intensity and decreased word duration (Griffin and Williams, 1987). Rothkrantz et al. (2004) used a variation on the Stroop-test, i.e., a gradual increase in difficulty by time-constraints (see Table 6). They found a significant increase in F0, F0-variation and a significant decrease in jitter. High frequency energy was, in contrary to the stated hypothesis, more present at a presentation delay of 2 and 2.5 s than the short delays at the end of the experiment (Rothkrantz et al., 2004). The Stroop-test was also used in a case study of Ruiz et al. (1996). This was the only study we encountered that observed large F0 increases, comparable with those during life-threatening emergency situations. A formant analysis showed that the effects on the formants F1, F2, and F3 were vowel specific. Finally, in a recent study (Huttunen et al., 2011), the speech of 13 military pilots was recorded during a simulator flight in which three levels of cognitive load were induced. The amount of cognitive load experienced by the subjects themselves was indicated on a visual analog scale. F0 significantly increased per load level, on average by 7 Hz with a larger increase at the most demanding cognitive tasks. Mean F0-range decreased by 5 Hz on average. The vocal intensity significantly increased by an average of 1 dB in function of cognitive load. The F0-intensity interrelation was controlled to verify that both increases were not due to noise. Lastly, in Johannes et al. (2007), which has been discussed earlier in the section of physical load, 11 participants performed several blocks of psychomotor tests alternated with relaxation. Time pressure and problem solving tasks evoked significant increases in F0 comparable with those found in the same study during physical activity within a tolerable level.

**Table 6:** Studies on the impact of cognitive load on voice and speech production.

Study	Speech process	Subjects	Context	Task	Speech measures	Results
Griffin and Williams, 1987	Phonation	*N* = 20	Laboratory	Psychomotor tests, increased difficulty.	F0 Amplitude in dB Word duration	Level 4 caused:
				• Level 1 counting from 1 to 10 for 10 times.		• Significantly increased F0 (106.95–118.91 Hz)
				• Level 2: psychomotor test (PMT) while counting 0–9.		• Increased intensity (49.38–57.12 dB).
				• Level 3: simple dichotic listening task (DLT) with vocalized responses.		• Decreased word duration (384.81–338.80 ms).
				• Level 4: combined DLT-PMT task.		
Hecker et al., 1968	Phonation	*N* = 5	Laboratory	Time-constraints in a read-aloud calculation task.	F0	Mixed results: increased and decreased F0-patterns.
Huttunen et al., 2011	Phonation	*N* = 13	Simulation flight	Flight simulation task with three levels of cognitive load: situation awareness, information processing and decision making.	F0 F0 range amplitude	• Increased F0 and amplitude in function of cognitive load.
						• Decreased F0 range.
Kurniawan et al., 2013	Phonation	Not mentioned – model testing	laboratory	• BL: questionnaire and 10 min relaxation.	F0 prediction models	No reliable predictive models in F0.
				• Low cognitive load: Stroop-Word congruent color test and easy mental-math test.		Voice stress is an individual dependent factor.
				• High cognitive load: Stroop-Word incongruent color test and hard mental-math tests.		
Johannes et al., 2007	Phonation	*N* = 11	Laboratory	Psychomotor tests: time pressure, problem solving test, sensorimotor coordination and a handgrip physical strength test) with alternated relaxation (nature images with composed music).	F0	• Increased F0 in time pressure and problem solving tasks.
Rothkrantz et al., 2004	Breathing Phonation	*N* = 108	Laboratory	Stroop-task with increased difficulty: shortening the time between the appearances of the presentation of the matched and non-matched color-word sample every minute with half a second.	F0 Jitter Duration	• Significant increased F0 (114.28–122.20 Hz) and F0-variation (7.36–10.11 Hz).
						• Significant decreased jitter (1.24–0.94%).
						• Significant decrease in jitter (1.24–0.94%).
						• High frequency energy more present in longer time-slots.
						• Decreased utterance duration.
Ruiz et al., 1996	Phonation	*N* = 1	Laboratory	Stroop-task.	F0 Formants F1, F2, F3	• Significant increase in F0 (127–164.8 Hz with a maximal peak value of 250 Hz).
						• Impact on formants vowel specific.

Also under cognitive load, multidimensional stress responses have been a topic of interest and this in order to develop fitness and training profiles of pilots (e.g., Simonov and Frolov, 1977; Simonov et al., 1980). Simonov and Frolov (1977) described a positive correlation between HR and F1 in space flight and preflight preparations. In another methodological study (Kurniawan et al., 2013), GSR and speech cues were equated in four different models on their accuracy to differentiate between cognitive load and recovery. The protocol contained a 1 h stress experiment with a baseline, low and high cognitive load. The authors (Kurniawan et al., 2013) found no reliable predictive models based on F0, concluding that voice stress remains an individual dependent factor.

#### Cognitive and Emotional Load

In a study of Brenner and colleagues that has been reported in an incomplete (*N* = 6) and complete analyzed set (*N* = 17) (i.e., Brenner and Shipp, 1988; Brenner et al., 1994), the impact of cognitive load with an emotional load factor of varying monetary incentives was investigated. Significant relations between cognitive load and F0 were shown. We will only report the results of the complete analyzed data set (i.e., Brenner et al., 1994). The subjects had to perform a computer-tracking task programmed on three to be accomplished difficulty levels with simultaneous ECG and speech monitoring. They found a significant increase in F0 (i.e., 2 Hz) and intensity (i.e., 1 dB) at the difficult task level in comparison with the easy task level. HR showed an interaction effect with the emotional load factor of reward or motivation. That is, in the difficult task, there was a large increase in HR when closer to the point of winning extra money. A decreased marginal effect was found on jitter, shimmer and speech rate (i.e., 4%) (Brenner and Shipp, 1988; Brenner et al., 1994). Furthermore, we found two studies (i.e., Tolkmitt and Scherer, 1986; Mendoza and Carballo, 1998) that attempted to disentangle cognitive and emotional load by including anxiety-trait parameters in the design. However, both papers used different anxiety scales and a different stress design. In Mendoza and Carballo (1998), the participants were 82 students that were classified as high, medium or low anxiety based on the STAI-Trait Anxiety Inventory. In a first high-stress experiment, it was intended to induce a high stress environment (i.e., the students were told that a bad performance on the task would result in a failure for the course). In a second low-stress experiment, the students were debriefed. The authors reported a similar impact of the induced cognitive load in the high versus low stressful environmental condition, i.e., increased F0, decreased jitter and shimmer and an increase in high-frequency harmonic energy (1600–4500 Hz) (Mendoza and Carballo, 1998). There was no influence of anxiety trait and no difference between the three STAI-groups. We found no methodological information with regard to jitter and shimmer. Tolkmitt and Scherer (1986) found interaction effects between cognitive load, individual anxiety traits and gender. Cognitive load was induced by easy and difficult logical reasoning tasks. Emotional load was induced by pictures of skin diseases (low emotional stress) and severe care accident injuries (high emotional stress). Out of 374 starting participants, 60 participants could be categorized in one of the three groups. Tolkmitt and Scherer (1986) found that male subjects with both low and high anxiety traits showed higher F0 values under cognitive load than under emotional load. Further, anxiety deniers, independent of gender, had higher mean F0 values under emotional stress than cognitive load. F0 floor increased under high emotional stress for high-anxious subjects and anxiety deniers, but decreased under high emotional stress for low-anxious subjects. With regard to formants, an interaction effect between gender and anxiety-trait was found. Only anxiety-denying women showed formant sensitivity to stress. They showed increased distance between F1/F2 and the neutral vowel frequency (i.e., the formant value when the vocal folds would be at rest) under high cognitive load and decreased distance between F1/F2 and the neutral vowel frequency under high emotional stress.

Finally, Scherer et al. (2002) distinguished between cognitive load and the subjective self-rated emotional load (i.e., the emotional state and intensity due to the cognitive task). The study involved 100 male participants (i.e., 25 native German, 16 native English, and 59 native French speakers). They found an increased speech rate and decrease in the decay and proportion of energy below 500 Hz under cognitive load. They found no impact of the reported stress-level. However, F0, significantly increased as a consequence of emotional load and not of cognitive load (see Table 7).

**Table 7:** Studies on the impact of mixed cognitive and emotional load on voice and speech production.

Study	Speech process	Subjects	Context	Task	Speech measures	Results
Brenner and Shipp, 1988; Brenner et al., 1994	Breathing Phonation	*N* = 8 (incomplete analysis) *N* = 17	Laboratory	Counting during a computer tracking task.	F0, speaking rate, amplitude, vocal intensity, vocal jitter and shimmer and a derived measure (combination of all of the speech variables except of vocal shimmer)	Only the results of Brenner et al. (1994) are reported.
						• Increased F0 (i.e., 2 Hz) and amplitude (i.e., 1 dB) at the difficult task level in comparison with the easy task level.
						• Decreased marginal effect on jitter, shimmer, and speech rate (i.e., 4%).
						• Amplitude and heart rate showed an interaction effect with the emotional factor of reward: increased heart rate when closer to the point of winning extra money.
Mendoza and Carballo, 1998	Phonation	*N* = 82	Cognitive load laboratory Emotional load real-life	Students were classified in high, medium, or low anxiety based on the STAI-Trait Anxiety Inventory	F0 Jitter, shimmer Harmonic energy	• Increased F0.
				• Backward reading of the alphabet.		• Decreased values in jitter and shimmer.
				• Tongue-twisters under time-pressure (with and without delayed auditory feedback).		• Increase in high-frequency harmonic energy (1600–4500 Hz).
				• sustained ‘a.’		
Scherer et al., 2002	Breathing Phonation	*N* = 100	Laboratory	• Psychomotor cognitive tests: time pressure, problem solving test, sensorimotor coordination; handgrip physical strength test alternated with relaxation periods.	Speech rate Energy below 500 Hz F0	• Increased speech rate in cognitive load but no impact of emotional load.
				• No particular inducement of emotional load, only a subjective self-rate.		• Decreased proportion of energy below 500 Hz during cognitive load but no impact of emotional load.
						• Increased F0 related to emotional load and not cognitive load.
Tolkmitt and Scherer, 1986	Phonation	*N* = 60 (male and female)	Both cognitive and emotional load: Laboratory	• Classification based on anxiety coping style (low anxiety, high anxiety and anxiety deniers) – based on the combination of the scores of the Manifestation Anxiety Scale (Taylor, 1953) and a Social Desirability Scale (Crowne and Marlow, 1964).	F0 F0 floor Formants F1/F2	• Male – low and high anxiety traits: higher F0 values under cognitive load than under emotional load.
				• Cognitive load: easy and difficult logical reasoning tasks.		• Anxiety deniers (both male and female): higher mean F0 under emotional than cognitive load.
				• Low and high emotional load: pictures of skin diseases/severe care accident injuries.		• F0 floor: increase in high emotional load in high-anxious subjects and anxiety deniers; decrease in high emotional load in low-anxious subjects.
						• Anxiety-denying women: increased distance between F1/F2 and decreased distance between F1/F2 in high emotional load.

## Discussion

In this review, we aimed to present a comprehensive overview on the use of VSA in the indication of physical, emotional, and cognitive load. Research on VSA has a variety of application potentials in function of different aims and goals. Therefore, in the result section, we commenced with an update of all of the parameters before reviewing the actual effect of the different types of load, physical, cognitive, and emotional as well as deleterious factors in human performance such as alcohol, sleep deprivation and hypoxia, on the voice output. We also encountered a large variety in methodological approaches and procedural differences in the chosen designs, parameters and stress inducers.

With regard to this miscellany of approaches, first of all, a clear distinction needs to be made between real-life and laboratory stress induction. It is notable that all of the studies that showed clear consensus in the impact of the stress effector were actually studies on real-life situations (Williams and Stevens, 1972; Kuroda et al., 1976; Streeter et al., 1983; Brenner et al., 1985; Mayer et al., 1994; Benson, 1995; Ruiz et al., 1996; Hansen and Patil, 2007; Demenko and Jastrzêbska, 2012), certainly when it comes to emergency situations (e.g., Kuroda et al., 1976; Brenner et al., 1985; Ruiz et al., 1996; Kirchhübel et al., 2011; Kurniawan et al., 2013). In real-life, emotional load has a tangible relevance and ecological validity not found in simulations since the individual is confronted with a real urge to provide in survival or at least well-being. To do so, one relies on an utilitarian function with a strong motivational factor that evokes intense and autonomic responses (e.g., the urge to remove from an object of fear or anger) (Scherer et al., 2004; Scherer, 2005; Kreibig, 2010). Secondly, it would be desirable to investigate structurally the period of stress induction that varies strongly over different studies. For instance, it has been claimed that to induce a reliable emotional or cognitive load under laboratory circumstances, an induction of 30 min would be required (Grassmann et al., 2017). Also Baker et al. (2008) argued that the length of physical load may have different outcomes on the final stress measured in the voice. Thirdly, although all of the studies conclude that VSA may be a promising stress detection tool, a large number of authors claim that VSA is highly limited by large interindividual differences. However, as stated by Grassmann et al. (2017), individual differences are often treated as errors or unexplained variability whereas it could be, on the contrary, treated as a signal instead of noise and thus function as a starting point to understand underlying mechanisms. From this perspective, inconsistency over studies may even be the result from ignoring the role or significance of individual differences (e.g., Cain, 2007; Grassmann et al., 2017). Therefore, we want to discuss the findings by using these interindividual differences as a window into potential psychophysiological mechanisms underlying both voice reactivity and the related stress-response.

### Phonation

#### F0 and F0-Range: A Vocal Mirror of the Equilibration Between Arousal and Top-Down Regulation?

The most extensively studied voice parameter we encountered was F0, which gives insight into the phonation component of voice production. Although F0 has regularly been considered as a universal stress indicator by a variety of authors (e.g., Tolkmitt and Scherer, 1986; Brenner and Shipp, 1988; Mendoza and Carballo, 1998), inconsistencies with regard to F0-reactivity have been reported as well (Hecker et al., 1968). Hecker et al. (1968) suggested, as a consequence of inconsistency in their study, that individual differences may be due to different manners to control a stress response. Indeed, in our review, we encountered a recurrent pattern over studies with regard to F0 and F0-variance, that may be understood in terms of the reciprocal interaction between bottom-up arousal and top-down regulation that characterizes a stress response (Thayer and Lane, 2000, 2009). We suggest that increased levels of F0 are linked with acute bottom-up processes of sympathetic arousal, whereas the narrowing of the F0-range or a decrease in F0-variability would express top-down regulation. The balance between both parameters may thus be mirroring the balance between bottom-up and top-down activity. With regard to cardio-respiration, variability and withdrawal have already become accepted features. Decreased HRV and respiratory variability are clearly associated with emotional (e.g., negative emotions such as anxiety, Boiten, 1998; Thayer and Lane, 2000; Wilhelm et al., 2001; Van Diest et al., 2006; Friedman, 2007) as well as cognitive load and top-down regulation (Thayer and Lane, 2000, 2009; Vlemincx et al., 2010, 2011). Knowing that breathing and the interconnectedness of the NV are the underpinnings of both cardiorespiratory and vocal events, it is not surprising that both systems appear to behave similarly toward stressors. Therefore, we want to highlight support for the fact that combined information of F0 and F0-range may give insight into the equilibration of bottom-up arousal and top-down regulation and as such the stability of the system [see also section “Final Conclusion and Research Agenda: Model for Voice and Effort (MoVE)”].

Firstly, the strongest F0-increases were found in life-threatening emergency situations (Williams and Stevens, 1972; Streeter et al., 1983; Brenner et al., 1985; Mayer et al., 1994; Ruiz et al., 1996) with frequencies that rose dramatically to levels sometimes redoubling the starting frequency (e.g., Williams and Stevens, 1972; Mayer et al., 1994). Moreover, along with these extreme mounting pitch levels, the F0-range appears to increase (Williams and Stevens, 1972; Streeter et al., 1983; Brenner et al., 1985; Mayer et al., 1994; Ruiz et al., 1996) as well, certainly in severe emergency circumstances such as just before a crash (Ruiz et al., 1996). Thus, at the point that top-down regulation silences, large F0-range increases occur. Secondly, in the studies of Streeter et al. (1983) and Ruiz et al. (1996), two communicators showed a different vocal output during emergency that can be related to the degree of top-down regulation expected in their role, position and training. The communicators without a specific top-down regulation training showed the typical pattern of increased F0 and F0-range during the first encounter of emergency, whereas those communicators that were trained to fulfill a mediating and regulating role during emergency, showed no increased or even decreased F0-ranges (Streeter et al., 1983; Ruiz et al., 1996). Moreover, as shown by the data of Ruiz et al. (1996), in the heat of the battle, when sympathetic arousal finally gained the upper hand and top-down regulation went off-line, F0-ranges increased after all (Ruiz et al., 1996). Thirdly, both fear (Demenko and Jastrzêbska (2012) and anger (Hansen and Patil, 2007; Demenko and Jastrzêbska, 2012) utterances as well as speech during exam stress (Sigmund, 2006) are characterized by increased F0 (Sigmund, 2006; Hansen and Patil, 2007; Demenko and Jastrzêbska, 2012), and an increased F0-range or variance (Sigmund, 2006; Hansen and Patil, 2007; Demenko and Jastrzêbska, 2012). Anger as well as fear are accepted to be amygdala-mediated emotions and behaviors that concur with sympathetic arousal in the need of prefrontal control when inhibition is desired (e.g., Morris et al., 1998; Whalen et al., 1998; Izdebski, 2008; see Kreibig, 2010 for a review on autonomic responses to emotions). The reported increased F0 and F0-range are thus a potential mirroring of a combined increased bottom-up arousal and decreased top-down regulation. Fourthly, in a study of Klingholz et al. (1988) on alcohol-intoxication, expanded F0-ranges were found as well. This is in line with the fact that alcohol consumption is known to switch activity from prefrontal toward subcortical limbic structures and thus to diminish top-down regulation (Volkow et al., 2008; Marinkovic et al., 2012). Fifthly, under cognitive load, F0-increases are also systematically reported (Griffin and Williams, 1987; Brenner and Shipp, 1988; Brenner et al., 1994; Ruiz et al., 1996; Mendoza and Carballo, 1998; Rothkrantz et al., 2004; Johannes et al., 2007; Huttunen et al., 2011) but they are clearly much less marked (with an exception of the participant in Ruiz et al., 1996) than during a life-threatening emergency [e.g., approximately 224 Hz during an extreme emergency (Williams and Stevens, 1972) against 11.96 Hz during laboratory induced cognitive load (Griffin and Williams, 1987)]. Moreover, cognitive load does not seem to increase the F0-range (Mendoza and Carballo, 1998; Johannes et al., 2007; Huttunen et al., 2011). Even on the contrary, Huttunen et al. (2011) reported a decrease in F0-range, despite an increase in the mean F0 level. This vocal output of a small F0-increase and a decrease in F0-range is in line with what has been described in the psychophysiological neurovisceral models as the ideal equilibration between bottom-up arousal and top-down regulation, i.e., a small arousal combined with increased cognitive control, to facilitate optimal cognitive performance (Lane, 2008; Thayer and Lane, 2009). A similar compromise between compensatory costs and effective performance has been defined as effort in the performance/cost trade-off model of Hockey (2013). According to Hockey (2013), a person’s psychophysiological output is the expression of the effort or the compensatory sources he/she has to tap to maintain the effectiveness of a given performance. Oppositely, in a person that is not motivated or not able to make an effort, the performance effectiveness will deteriorate but the strain of the compensatory regulation on the system will be less as well. Indeed, increased HR, BP, (nor)adrenaline and decreased HRV have been regularly reported as compensatory regulation or effort to maintain successful performance in several studies (e.g., Tattersall and Hockey, 1995; Tafalla and Evans, 1997; Pattyn et al., 2008; Hockey et al., 2009; Thayer and Lane, 2009; Grassmann et al., 2017). On a vocal level, though, the findings of the current review showed that the output of an extreme burst of effort -such as during a life-threatening condition- was characterized by a dramatic F0-increase. The inability to make an effort should thus logically result in a decrease of F0. A typical condition in which people gradually lose the ability to sustain both effort and cognitive performance (e.g., Pilcher and Walters, 1997) is when they are sleep deprived. Indeed, in Whitmore and Fisher (1996), a F0-decrease in combination with diminished arousal and impaired cognitive performance at the low-circadian time-points during a 36 h cycle of sleep-deprivation was reported. However, it has not yet been examined whether the voice output is a circadian phenomenon as such, independent from sleep deprivation, or whether it changes in function of the circadian rhythm only under load of sleep deprivation. Furthermore, unfortunately, studies on the impact of sleep deprivation on voice events did not report any data on the F0-range. An increase in F0-range would be expected since sleep deprivation has been connected with a failure of top-down regulation documented by decreased functional connectivity between prefrontal cortical regions and limbic activity in the amygdala (Yoo et al., 2007). An extreme and particular form of effort-depletion appears to occur during acute hypobaric hypoxia (Saito et al., 1980). Hypoxia can be considered as a condition of cognitive breakdown (Petrassi et al., 2012) in which mechanisms of effort are damaged (Garner et al., 1990; Caquelard et al., 2000) ending in a totally impaired performance (Petrassi et al., 2012). Nevertheless, acute hypobaric hypoxia is known to activate sympathetic arousal (e.g., Hainsworth et al., 2007; West et al., 2007; Petrassi et al., 2012). The cardiac and pulmonary output surge in order to supply the vital organs, muscles and the brain of oxygen (Hainsworth et al., 2007; West et al., 2007; Petrassi et al., 2012) until paralysis as the foreboding of unconsciousness will appear (West et al., 2007). This means that, although arousal is extremely high, the compensatory regulation of effort appears to be inadequate to avoid deteriorated performance. On a vocal level, the voice output appears to respond to the effort depletion rather than the sympathetic arousal. Saito et al. (1980) reported a decrease in mean F0 of 45.2 Hz in response to acute hypobaric hypoxia. Moreover, the general withdrawal of central motor components shows up in a deteriorated articulatory system with increased VOT (Saito et al., 1980). Possibly, the complex laryngeal muscle system of the voice is one of the first systems vulnerable to the effects due to acute hypoxia, notwithstanding the effort of the body to counteract the oxygen loss by means of hyper sympathetic activity. Noteworthy, although more studies are needed in this research domain, VOT appears to have the potential to differentiate between acute and chronic hypoxia, since it was reported to increase in the first and decrease in the latter. If this is the case, this early stage voice reactivity could be interesting in safety prevention contexts.

We did not encounter studies including the rate of F0 change (e.g., Nilsonne et al., 1988) or the direction of F0 change (e.g., Isaacs and Watson, 2010) as a measure of interest. These measurements are based on the combination of both F0 and time variables with the slope providing extra information on the direction of the F0 course. These variables were successfully applied in early studies on vocal characteristics in clinical depressed persons (Nilsonne, 1987; Nilsonne et al., 1988) and accent detection during speech (Isaacs and Watson, 2010) and may provide additional information with regard to the acuteness of the evolvement of a stress response.

#### Jitter, a Hypothetical Prosody-Independent Mirror of Bottom Up Arousal

During phonation, stress can also induce small variations or asymmetries in the tension of the cricothyroid muscle (Brenner and Shipp, 1988) and/or fluctuations in subglottal pressure (Yao et al., 2016). These voice inconsistencies or ‘noise’ in the voice are measured in terms of jitter, shimmer, SNR/HNR reactivity and HRF (e.g., Clark and Yallop, 1990; Dietrich and Abbott, 2012; Boersma and Weenink, 2013) and are sometimes descriptively referred to as ‘hoarseness’ (e.g., Protopapas and Lieberman, 1997, p. 2267; Postma-Nilsenová et al., 2016, p. 1349). Intuitively, one would expect that hoarseness would increase under stress. However, studies found that acute stressors decrease jitter and shimmer. Interestingly, in accordance with the findings in F0 parameters, the impact on jitter and shimmer seems to be larger during emotional load in an emergency (Brenner et al., 1985) than during cognitive load (Brenner and Shipp, 1988; Brenner et al., 1994). In the studies on cognitive load of Brenner and colleagues, for instance, only marginal decreases in jitter and shimmer were found. Brenner and Shipp (1988) hypothesized a direct negative correlation between the level of jitter and that of stress. In Mendoza and Carballo (1998), both cognitive and combined cognitive and emotional load (i.e., the threat to students that a bad cognitive performance would result in failure of the course) decreased jitter and shimmer significantly. Although Mendoza and Carballo (1998) argued that there were no differences between the reactivity patterns of the pure cognitive load condition and the added emotional load condition, their tables show a clearly larger impact when emotional load was induced. However, unfortunately, this difference between the non-emotional and emotional load group was not tested. here was only one study that found increased jitter during moments that were selected as stressful during a conversation between a medical doctor and his patient (Postma-Nilsenová et al., 2016). Oddly, the authors argue that their results are in correspondence with the general findings on stress and jitter, referring to a review (Giddens et al., 2013) in which actually the inverse result, i.e., decreased jitter in response to a stressor, is reported as a stress-related voice parameters. Postma-Nilsenová et al. (2016) controlled the occurrence of stress by GSR. Nevertheless, another study that also controlled for stress by means of ECG-registration, found -in correspondence with the former studies- decreased jitter. The different outcomes may be due to methodological issues in Postma-Nilsenová et al. (2016). There was no information with regard to the chosen GSR-analysis (i.e., peak-detection or continuous interval measuring), the so called ‘SC interval’ (i.e., with regard to its time period) and the jitter analysis (whether vowels were selected and in what manner). Moreover, the authors did not take a GSR post-stimulus 1–3 s or 1–5 s latency window into account (Dawson et al., 1990; Benedek and Kaernbach, 2010). Therefore, it is not sure whether the time-constants of the measured stress-periods and voice utterances actually matched.

#### The Competition for Resources Between Emotional and Cognitive Load: A Potential Role of the ACC

The overall findings with regard to vocal fold reactivity during phonation in response to emotional and cognitive load could be summed up with the perspective from Bishop (2009) who found that there is an interaction between emotional and cognitive load that is the result of a competition for resources. It has been hypothesized that, when coping with anxiety during cognitive load, top-down cognitive control mechanisms compete for resources with bottom-up emotional sensory mechanisms (e.g., Bishop, 2009; King and Schaefer, 2010). This idea has been elaborated in subsequent studies that showed that the influence of anxiety on cognitive load is more pronounced in conditions of low cognitive load and high anxiety than high cognitive load (e.g., Vytal et al., 2012). Higher-demand tasks and top-down cognitive control would reallocate resources toward the task demands and thereby reduce the influence of high-arousal anxiety (King and Schaefer, 2010). In other words, high cognitive demands and the individual’s capacity to cope with these demands, maintains top-down cognitive control and diminishes the chance for anxiety to intervene. The output of the balance between emotional and cognitive load is an integration of emotional processing, executive control of attention and psychomotor processes in the final performance (e.g., Benarroch, 1993; Lane, 2008).

This equilibration between arousal and top-down regulation has been suggested to occur in the ACC (Lane, 2008). This region is involved in vocal activity as well (e.g., Vogt and Gabriel, 1993; Paus, 2001). The vocal apparatus is connected with both the bifurcated pathways of the NV and the ACC. On the one hand, the superior laryngeal nerve, that stems from the NV, is known to innervate the cricothyroid muscle that is involved in vocal fold stretching and pitch regulation (Kreiman and Sidtis, 2011). On the other hand, the executive pathways of the ACC to the brainstem nuclei involved in the ‘fight and flight modus’ (Cacioppo et al., 2007) and its top-down regulation (e.g., Benarroch, 1993; Thayer and Lane, 2000, 2009; Lane, 2008) are also part of particular pathways that execute psychomotor (Lane, 2008) and vocal (e.g., Vogt and Gabriel, 1993; Paus, 2001) behavior. Possibly, the connection between the ACC to the autonomic circuit of the NV and further connections to laryngeal nerves may serve as a major route to voice-stress output during cognitive and emotional load.

### Breathing

#### Physical Load and the Competition for Ventilation Processes

**FIGURE 1:**
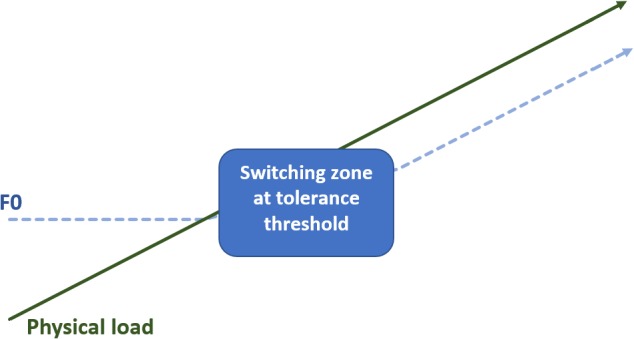
Switching zone of voice reactivity to physical load. As a consequence of a competition for ventilation processes, F0 increases in response to physical load from the point that this load is not well tolerated anymore (e.g., Johannes et al., 2007).

The impact of physical load on voice production is also the result of a competitive mechanism. However, this competition for resources has place on the level of the breathing component of voice production. With regard to emotional and cognitive load, the largest impact on the voice output interfered with the phonation component, giving insight into the respective balance between both types of load. With regard to physical load, the speech process is part of the competition itself. Voice perturbations during physical activity are said to be due to an internal competition between the ventilation processes required to speak and those to meet the metabolic demands of the exercised muscles, which primarily results in a pattern of appropriate breathing pauses (e.g., Otis and Clark, 1968; Bunn and Mead, 1971; Phillipson et al., 1978; Doust and Patrick, 1981; Meckel et al., 2002; Baker et al., 2008; Rodríguez-Marroyo et al., 2013). However, physical load does not impact the voice as long as this load is well tolerated by the subjects (Mohler, 1982; Johannes et al., 2007). With regard to F0, in Johannes et al. (2007), there were no significant F0-increases at a moderate level of physical activity between 100 and 200 W, although there was a significant impact on HR and BP. Moreover, the F0-range even decreased at that point. Research comparing the impact of speech versus non-speech on cardiorespiratory events during physical activity (Doust and Patrick, 1981; Meckel et al., 2002; Baker et al., 2008) may bring insight into this ‘delayed switching zone of voice-reactivity’ to physical load. When someone talks in an early stage of physical exertion, the human body appears to foresee in the maintenance of ventilatory needs to serve voluntary speech, even at the cost of VO_2_ uptake (e.g., Baker et al., 2008). In this stage, the voice gains an advantage over the other metabolic needs in their ventilatory competition and ventilation and VO_2_ decrease. However, when physical load continues, the body seems to reset in order to be able to cope with these long-term physical demands, this time at costs of the voice-production system that will be robbed of its strengths. So, as long as physical load is tolerated well, ventilation and oxygen consumption can be partly hijacked by the speech system. However, when physical load becomes more intense, oxygen and ventilation reservoirs need to restore. At this point, both ventilation and VO_2_, along with HR start to re-increase toward the earlier levels measured during non-speech (Doust and Patrick, 1981; Meckel et al., 2002; Baker et al., 2008) (see also Figure 1).

#### F0-Range of Variance, Fatigue and Motivational Top-Down Regulation

It is interesting that during tolerable levels of physical load, reduced F0-range values were reported (Johannes et al., 2007). Maintaining a similar reasoning to earlier on, this could point to top-down regulation processes in the attempt to exert control. Already in 1986, Hockey, pointed to the influence of top-down components in physical fatigue with regard to muscle exertion. Hockey (1986) showed that the supposed point of exhaustion and someone’s experience of fatigue can differ due to a cognitive motivational factor. In his study, participants decided that muscular effort was no longer possible well before the physiological point of exhaustion had been reached. However, this motivational component can work in the opposite manner as well. Marcora and Staiano (2010) showed that -when highly motivated- subjects can produce immediately after a physical point of exhaustion a maximal voluntary cycling power, three times stronger than the power output required by the time to exhaustion. These studies are illustrative of the fact that physical exhaustion may be a mental phenomenon rather than a pure dose-response physical metabolic failure to a task (Pattyn et al., 2018). According to Marcora and Staiano (2010), exhaustion is the result of either a motivational factor (i.e., one decides to not further invest or give up) or a perceived inability (i.e., effort is too high to continue) rather than a simple (physical) failure to the task. So, as with in top-down regulation during cognitive load, top-down regulation during physical activity may concur with a more controlled voice as well, expressed by a narrowing F0-range. Moreover, it would not be implausible to hypothesize that people are more accustomed to master their voice under physical load than under cognitive or emotional load since the first is part of common day life. Support for this can be found in the fact that, for instance, during cognitive load, although the F0-range narrows, the mean F0 itself starts to rise, which we did not encounter in the studies on physical load. Moreover, with regard to emotional load, a reduction in F0-range only concurred with a decrease in F0 in populations recognized for a high trained emotion regulation (e.g., pilots) (Ruiz et al., 1996). So, with regard to physical load, F0 voice parameters probably rather respond to a cognitive component than obeying a metabolic dose-response relationship. The fact that the voice responds to this zone of exhaustion has already shown its usefulness for physical sport and exercise training procedures. The relationship between speech and ventilatory processes during exercise is the basic principle of the Talk Test (TT). In the TT, it is stated that the point on which a healthy, homogenous and well-trained person cannot speak aloud anymore during exercise is a marker of the ventilatory threshold to be respected during training (Dehart-Beverley et al., 2000; Shafer et al., 2000; Recalde et al., 2002; Persinger et al., 2004; Foster et al., 2008, 2009; Norman et al., 2008; Jeans et al., 2011; Rodríguez-Marroyo et al., 2013).

### Resonance

#### Formant Reactivity as a Hypothetical Vocal Mirror of the Equilibration Between Arousal and Top-Down Regulation

Whereas muscle activity of the larynx and vibrating vocal cords affects the F0 and thus intonation, the muscles involved in the shaping of the resonant cavities of the vocal tract system do not impact the F0 but are involved in further quality of sound shaping and vowel and consonant pronunciation (Gopalan et al., 1999). Their activity can be obtained by formant-analyses and MFCC. Although Klingholz et al. (1988) argued that formant analysis is a poor stress discriminator, also here, some logical agreements over the reviewed studies may be present. Maintaining the hypothesis that voice reactivity may differentiate bottom-up arousal and top-down regulation, we found comparable recurring patterns in the formant reactivity. That is, bottom-up arousal may cause increased F2 and decreased F1/F2-ratio and top-down regulation may be related with decreased F3 and increased F1/F2 ratio. Firstly, in Ruiz et al. (1996), the increased F0 and F0-range in the pilot’s voice during high emotional load concurred with increased F2, whereas in the voice of the top-down regulating co-pilot, a decrease in F3 was observed (Ruiz et al., 1996). An increased F2 was also reported in other studies during emotional load. For instance, in Hansen and Patil (2007), increased F1, F2, and F3 was observed in response of anger. According to Hansen and Patil (2007), F1 and F2 appears to increase in general under emotional load. Moreover, we suggest that an increase or decrease of F3 may be related with a respective diminished or ameliorated level of top-down self-regulation or cognitive control. Also supportive of a similar increasing F2-reactivity under emotional load, were the reports of Tolkmitt and Scherer (1986) showing that high emotional stress, in anxiety-denying women, was marked by a decreased difference in distance between F1 and F2. In the same study and same population, high cognitive load was marked with an increased F1–F2 difference. As stated by Tolkmitt and Scherer (1986), increased F1–F2 differences reflect a frequency movement away from the neutral formants and toward the target formants pointing to a more accurate and controlled articulation. Plausibly, increased accurate articulation under cognitive load may be indicative of an effort to master speech in a more controlled manner. Certainly, when taking into account that the subjects that showed increased F1–F2 difference were anxiety denying. An anxiety-denying trait is known to be characterized by high cognitive top-down control (Lane, 2008) to suppress the underlying anxiety (Ketterer et al., 2004), comparable with the mechanism demonstrated in the study of Vytal et al. (2012) that showed the competition between anxiety and cognition resources. Moreover, it is known that the F1–F2 distance increases with an elevated position of the larynx (Sundberg and Nordström, 1976) which can be caused by, among others, laryngeal musculoskeletal tension related to stress-reactivity (Roy and Bless, 2000). Notably—in support of the above hypothesized top-down inhibition—voice problems related to an elevated position in the larynx have been related to introvert personalities (Roy and Bless, 2000; Van Houtte et al., 2011) who are reported to control their arousal by inhibitory behavioral and physiological (Gray, 1970) but also vocal laryngeal (Roy and Bless, 2000) regulation systems.

### Physiological Characteristics Complementary to Acoustical Characteristics

In the above described top-down/bottom-up equilibration during stress exposure, the impact of some physiological characteristics should be taken into account when studying voice stress factors, certainly in real-life urgency circumstances where increased F0 and vocal loudness are common speech factors. As explained in the introduction, they are both related with subglottal pressure (we refer the interested reader to Herbst et al., 2015; Zhang, 2015; Yao et al., 2016; Sundberg, 2017). Moreover, also the chosen speech phonation type or degree of glottal adduction is related with both subglottal pressure (Herbst et al., 2015) and NAQ (Godin and Hansen, 2015). Hence, it is necessary to take potential physiological dynamics into consideration when interpreting acoustical characteristics.

### Final Conclusion and Research Agenda: Model for Voice and Effort (MoVE)

**FIGURE 2:**
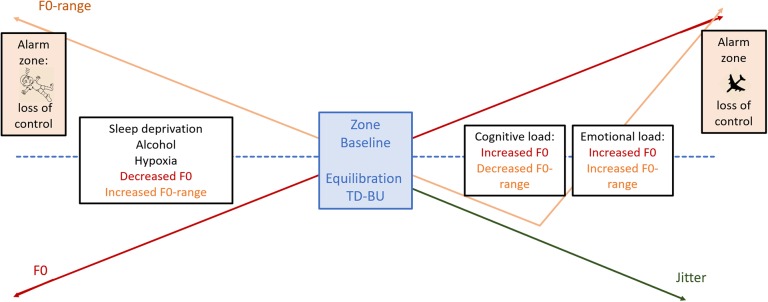
“Model for Voice and Effort” (MoVE). The MoVE shows how the activity of ongoing top-down (TD on the Figure) and bottom-up (BU on the Figure) processes are mirrored within the phonation voice parameters F0, F0-range and jitter. Increased F0-ranges correspond with reduced top-down processes reaching an alarm zone when cognitive top-down control is lost—such as in life-threatening emergency situations (e.g., flight crash, alcohol intoxication). Decreased F0-ranges are consequential of high cognitive load and top-down control. The additional information of mean F0 values gives insight in the bottom-up arousal activity and the effort a subject is capable to generate. Highly increased or decreased F0-values are indicative of effort-depletion, also reaching an alarm-zone in life-threatening emergency situations (e.g., flight crash, alcohol intoxication). Jitter expresses bottom-up arousal in an inverse manner. Cognitive and emotional load correspond with respective small and larger reduced jitter values.

When treating interindividual differences as a signal in place of noise, a large part of the inconsistencies may be explanatory for underlying mechanisms of the balance between top-down and bottom up processes during different types of loads. As illustrated in the MoVE (see Figure 2), the respective activity of ongoing top-down and bottom-up processes dependent from the situation and a person’s resource management finds a way out in the phonation voice parameters F0, F0-range and jitter. Increased F0-ranges correspond with reduced top-down processes reaching an alarm zone when cognitive top-down control is lost—such as in life-threatening emergency situations (e.g., flight crash, alcohol intoxication)—whereas decreased F0-ranges are measured in a situation that demands high cognitive load and top-down control. The additional information of mean F0 values and jitter give insight in the bottom-up arousal activity and the effort a subject is capable to generate. Highly increased or decreased F0-values are indicative of effort-depletion, also reaching an alarm-zone in life-threatening emergency situations and jitter expresses bottom-up arousal in an inverse manner.

Taking into consideration the findings of the current review, it seems that the application of VSA could be fine-tuned by a structural research agenda that takes into account the following factors. A clear distinction needs to be made between the induction of load in real-life versus laboratory conditions (Tolkmitt and Scherer, 1986; Kreibig, 2010; Pattyn et al., 2010; Grassmann et al., 2017). The current review supported that real-life emergency situations have a much stronger impact on the voice output than a laboratory induced load. Related to this issue, the duration of load induction needs to be examined within each type of stressor. Additionally, as pointed by Godin and Hansen (2015) and as shown in the approach of the current review, it is important to not focus on the expression of each voice parameter separately but to have attention for combined patterns of several voice parameters that may respond in a simultaneous meaningful manner. Nevertheless, potential separate physiological reactions in glottal parameters should be considered in final interpretations (Herbst et al., 2015; Sundberg, 2017). Furthermore, to obtain a systematical overview, it would be desirable to develop a set of standardized speech samples that could be used independently from language. The studies we encountered in the current review were all conducted in different languages, sometimes even not the native language (Kiss et al., 2014) or with mixed languages within one study (Scherer et al., 2002). Obviously, each language has its particular constellations of vowels, consonants, formants and breathing patterns (e.g., Dietrich and Abbott, 2012; Eklund, 2015) which may create already a difference in the outcome on a baseline level. Finally, it is remarkable, that although voice stress is a pure psychophysiological phenomenon in which the expiratory breathing phase is a regulatory factor, a psychophysiological approach that includes the measuring of respiration is not existing. Respiration is the driving force of both the processes of stress (e.g., Berntson et al., 2007; Pattyn et al., 2010; Vlemincx et al., 2010, 2011; Grassmann et al., 2017) and voice production (e.g., Dietrich and Abbott, 2012) and may form the missing link to fully understand the underlying mechanisms of the dynamic between speech and stress.

## Author Contributions

MVP literature research and writing. XN literature research and technical discussions. FM content discussion and proofreading. NP literature research, conceptual/content discussions, and content and proofreading.

## Conflict of Interest Statement

The authors declare that the research was conducted in the absence of any commercial or financial relationships that could be construed as a potential conflict of interest.
